# Ring of Hope: A Novice’s Experience in a Cancer Centre

**DOI:** 10.1007/s13187-023-02346-0

**Published:** 2023-07-31

**Authors:** Muhammad Hamza Shah

**Affiliations:** School of Medicine, Dentistry & Biomedical Sciences, Queen’s University Belfast, Belfast, UK

**Keywords:** Bell ringing, Cancer survivorship, Cancer journey, Psycho-oncology

## Abstract

The ringing of a bell has become an honored and cherished tradition in the realm of cancer survivorship care. As a medical student, I was initially unaware of this practice, but I have since gained a profound appreciation for its deep significance and emotional potency. Through the act of ringing the bell, patients who have successfully completed their cancer treatment are able to mark the end of a gruelling journey, while also heralding the start of a new and hopeful chapter in their lives.

In recent years, the tradition of bell ringing has become a beloved and celebrated custom in the world of oncology and cancer survivorship care. The concept is rooted in the notion that when a patient successfully completes their cancer treatment, they are invited to ring a bell to symbolize the end of their battle and the beginning of their journey as a triumphant cancer survivor [1]. As a medical student on my oncology placement, I was unfamiliar with this tradition, and witnessing it on my first day was a truly remarkable experience. As I entered the ward, I saw this middle-aged woman make her way through the main corridor alongside a group of nurses and her doctor. With each step, it seemed that she grew more hopeful, smiling at those around her, knowing that she was about to ring the bell that would signify her triumph over cancer. As the group approached the bell, the woman reached out with a fierce determination, grasping the cord with a palpable sense of purpose.

With each tug, the sound of the bell filled the air, reverberating with a power that spoke to the strength and resilience of the human spirit. In that moment, I realized that the act of ringing the bell was not just a simple tradition, but a profound and deeply meaningful symbol of hope and triumph over adversity. It was a reminder that even in the darkest moments, there is always the potential for light and the possibility of a brighter tomorrow. The ringing of the bell was a testament to the indomitable spirit and steadfast resilience of cancer patients, a reminder of the unwavering support they receive from their loved ones and the dedicated healthcare professionals. Yet, it was also a poignant reminder of the profound emotional toll that cancer takes on individuals and their families, a bittersweet reminder that each ring of the bell encapsulates a singular and intensely personal odyssey of anguish, trepidation, and optimism.

As I watched the middle-aged woman triumphantly ring the bell, I couldn't help but think of the countless others still undergoing treatment, looking on with both hope and longing. For those in the midst of their cancer journey, witnessing the bell ringing ceremony can be a source of both inspiration and pain, as they are reminded of the difficult road ahead, yet also filled with a sense of hope that one day, they too will ring that bell. Conversely, the bell ringing ceremony can also be a double-edged sword, evoking both hope and fear. It serves as a stark reminder of the challenges that lie ahead and it can be a painful reminder of the struggles they are still facing.

Nevertheless, the need for cancer having an outlet to help them cope with the physical and emotional challenges of their disease is heavily recognized. Much of the research on this topic focuses on this outlet being present during the cancer journey but starting the cancer-free journey also requires adequate support and resources to help survivors navigate their new normal [2]. The ringing of the bell marks the end of treatment, but it's just the beginning of a new journey for cancer survivors. It's crucial that they have access to ongoing care, support groups, and resources to help them transition back into their daily lives. By continuing to raise awareness funding for cancer research, we can work towards not only finding a cure but also providing long-term support for survivors.
